# A single amino acid substitution determines susceptibility of *Clostridioides difficile* toxin B variants to monoclonal antibody neutralization

**DOI:** 10.3389/fcimb.2026.1849926

**Published:** 2026-05-28

**Authors:** Kerisa Hall, Ishrya Sharma, Shasika Jayarathne, Hiba Osmani, Drishya Sudarsanan, Shannon Moonah

**Affiliations:** 1 Department of Medicine, University of Florida, Gainesville, FL, United States; 2 College of Medicine, University of Vermont, Burlington, VT, United States; 3 Department of Molecular Genetics and Microbiology, University of Florida, Gainesville, FL, United States

**Keywords:** amino acid substitution, antibody, C. difficile, cell death, therapeutics, variants

## Abstract

Monoclonal antibodies have become an integral component of modern therapeutics, however, their efficacy can be diminished by variation in target proteins, emphasizing the need to define these changes to guide the development of effective therapies. In this brief report, we uncover the molecular mechanism underlying a key limitation of bezlotoxumab, a monoclonal antibody targeting *Clostridioides difficile* toxin B (TcdB), specifically its reduced neutralizing activity against certain TcdB variants. We show that four major toxin B variants, B1–B4 (TcdB1–TcdB4), account for approximately 99.9% of circulating *C. difficile* strains globally. Bezlotoxumab neutralized TcdB1 and TcdB3 mediated cell death in human colonic epithelial cells, but not TcdB2 or TcdB4. Sequence analysis revealed that TcdB1 and TcdB3 share a conserved residue within a key antibody-neutralizing region, containing glutamic acid at position 2033 (E2033), whereas TcdB2 and TcdB4 contain an alanine at this position (A2033). Substitution of A2033 with E2033 in TcdB2 restored susceptibility to antibody-mediated neutralization. These findings help define the molecular basis of reduced susceptibility among toxin B variants and may guide the design of next-generation vaccines and antibody-based therapeutics against *C. difficile*.

## Introduction

*Clostridioides difficile* infection, first identified as the cause of antibiotic-associated colitis in 1978, is now the leading healthcare-associated infection in the United States, contributing to substantial morbidity and mortality, with an estimated 500,000 infections and 29,000 deaths annually (Guh et al., 2020; Johnson et al., 2021). Despite current therapies, recurrent infections occur in up to 30% of patients making it a priority to develop novel strategies to treat and prevent *C. difficile* infection (Hopkins and Wilson, 2018; Guh et al., 2020; Johnson et al., 2021; Shirley et al., 2023). *C. difficile* infection is a toxin-mediated disease in which the bacterium secretes exotoxins in the gut that induce intestinal epithelial cell damage, leading to inflammatory colitis (Mansfield et al., 2020; Mileto et al., 2020; Markham et al., 2021; Tornel et al., 2024). Toxin B (TcdB) induces cell death and *C. difficile* strains lacking TcdB do not cause disease (Lyras et al., 2009; Steele et al., 2013; Wilcox et al., 2017). Antibody therapy targeting TcdB reduces recurrence and disease severity, whereas antibodies against toxin A (TcdA) do not provide additional benefit (Lowy et al., 2010; Wilcox et al., 2017; Tornel et al., 2024; Peritore-Galve et al., 2025). Thus, targeting TcdB represents a significant therapeutic strategy for *C. difficile* infection.

The monoclonal antibody bezlotoxumab was approved for the prevention of recurrent *C. difficile* infection but was recently discontinued, eliminating an entire therapeutic option and highlighting the continued need for new therapies. Bezlotoxumab binds to TcdB and prevents its interaction with host cells, thus inhibiting toxin-mediated cell death (Wilcox et al., 2017). However, TcdB exists as multiple variants that differ in amino acid sequence, and a major limitation of bezlotoxumab is its reduced neutralizing activity against certain toxin B variants (Chen et al., 2021). Given that there are ongoing efforts to develop antibody-based therapies against TcdB, defining the molecular basis by which antibodies neutralize some variants but not others help reveal mechanisms of variant escape from neutralization, identifies vulnerable versus resistant epitopes, and guides the design of improved therapies. These insights may ultimately allow for the development of more clinically effective treatments that account for variant diversity. Here, we sought to identify the molecular basis for the lack of response of bezlotoxumab against certain toxin B variants and found that a single amino acid substitution determines susceptibility to antibody-mediated neutralization.

## Methods

### Generation of human organoid-derived colonic monolayers

Human colonic organoids and monolayers were created from human induced pluripotent stem cells (hiPSC) and maintained in culture with STEMdiff™ Intestinal Organoid Growth Medium and IntestiCult™ Monolayer Growth Medium, respectively (Stemcell Technologies) as per manufacturer’s protocol.

### Toxin-induced cytotoxicity assay

Human colonic epithelial cells were treated with TcdB (10 ng/mL), control antibody, or bezlotoxumab (5 μg/mL) for 18 hours. TcdB was pre-incubated with bezlotoxumab (No. PIMA541962; Invitrogen) or control antibody in culture media at 37 °C for 1 hour prior to addition to the cells to maximize neutralization. The toxin-antibody mixture in media was then added to the cells. Following treatment, cells were stained with annexin V and propidium iodide (No. 640914; BioLegend) and analyzed for cell death by flow cytometry (Riccardi and Nicoletti, 2006; Tornel et al., 2024) using Cytek Aurora flow cytometer (Cytek).

### Immunoblotting

For immunoblotting, samples were separated on polyacrylamide gels and then transferred onto nitrocellulose membranes (Bio-Rad). Blots were blocked with bovine serum albumin, followed by incubation with the following antibodies: bezlotoxumab recombinant human monoclonal (No. PIMA541962; Invitrogen), procaspase 3 and cleaved (active) caspase 3 (No. 9662S and No. 9664S, respectively; Cell Signaling Technology), and actin (No. A2066; Sigma) at 4 °C. Blots were then incubated with horseradish peroxidase conjugated anti-rabbit (No. A0545; Sigma) or anti-human (No. ab87422; Abcam) IgG secondary antibodies. Enhanced chemiluminescence (No. 32106X4; Thermo Scientific) substrates were used to detect HRP activity.

### Generation of TcdB variant constructs

TcdB1 was expressed and purified as previously described in (Yang et al., 2008; Tornel et al., 2024). The TcdB2 gene sequence was codon optimized for expression in *Bacillus megaterium* using the IDT Codon Optimization Tool. Restriction enzyme sites compatible with the pHis1522 expression vector were added to the optimized sequence, including BsrGI at the 5′ end and SphI at the 3′ end, with a 6×His tag inserted upstream of the 3′ site. Internal restriction sites were removed through silent mutations based on *Bacillus* codon usage. For cloning feasibility, the gene was divided into two fragments at an internal KpnI site. For cloning, Fragment 1 was ligated into the pHis1522 vector using BsrGI and KpnI, and the ligation product was transformed into competent cells for selection on ampicillin plates. Positive colonies were identified by colony PCR. Fragment 2 was subsequently inserted by digesting both the pHis1522 vector with fragment 1 and the second fragment with KpnI and SphI, followed by ligation using T4 DNA ligase and transformation. For TcdB3 and TcdB4, a similar two-fragment cloning strategy was used. TcdB3 fragments were cloned using BsrGI/BglII (fragment 1) and BglII/BamHI (fragment 2), while TcdB4 fragments were cloned using BsrGI/BamHI (fragment 1) and BamHI/SphI (fragment 2). Constructs were ligated into the pHis1522 vector. Correct assembly of the full-length construct of all variants was confirmed by sequencing.

### Computational analysis

The sequence of Bezlotoxumab was obtained from the KEGG Drug Database (Kanehisa et al., 2017). The three-dimensional structure of the antibody was predicted using ABodyBuilder2 (Abanades et al., 2023). The structure of *Clostridioides difficile* toxin B (TcdB) was predicted using the AlphaFold Server (Abramson et al., 2024), using residues 1835–2100 of the CROP domain (Orth et al., 2014). Protein–protein docking between bezlotoxumab and TcdB was predicted using pyDockWeb to model interactions as this approach emphasizes electrostatic and van der Waals interactions (Jiménez-García et al., 2013; Rodríguez-Lumbreras et al., 2025), which have been shown to contribute to this antibody–antigen interaction (Hernandez et al., 2015; Karnchanapandh et al., 2024). The resulting models were then analyzed using Residue Interaction Network Generator (RING) 4.0 to identify and characterize residue-level interactions between the antibody and toxin (Del Conte et al., 2024).

### Analysis of TcdB variant prevalence

The global prevalence of *Clostridioides difficile* toxin B variants was determined using subtype data from human isolates from (Shen et al., 2020).

### Statistical tests

Statistical differences were determined using unpaired two-tailed t-test. A p value less than 0.05 was considered statistically significant.

## Results

### Susceptibility to antibody-mediated neutralization varies among TcdB variants

**Figure 1:**
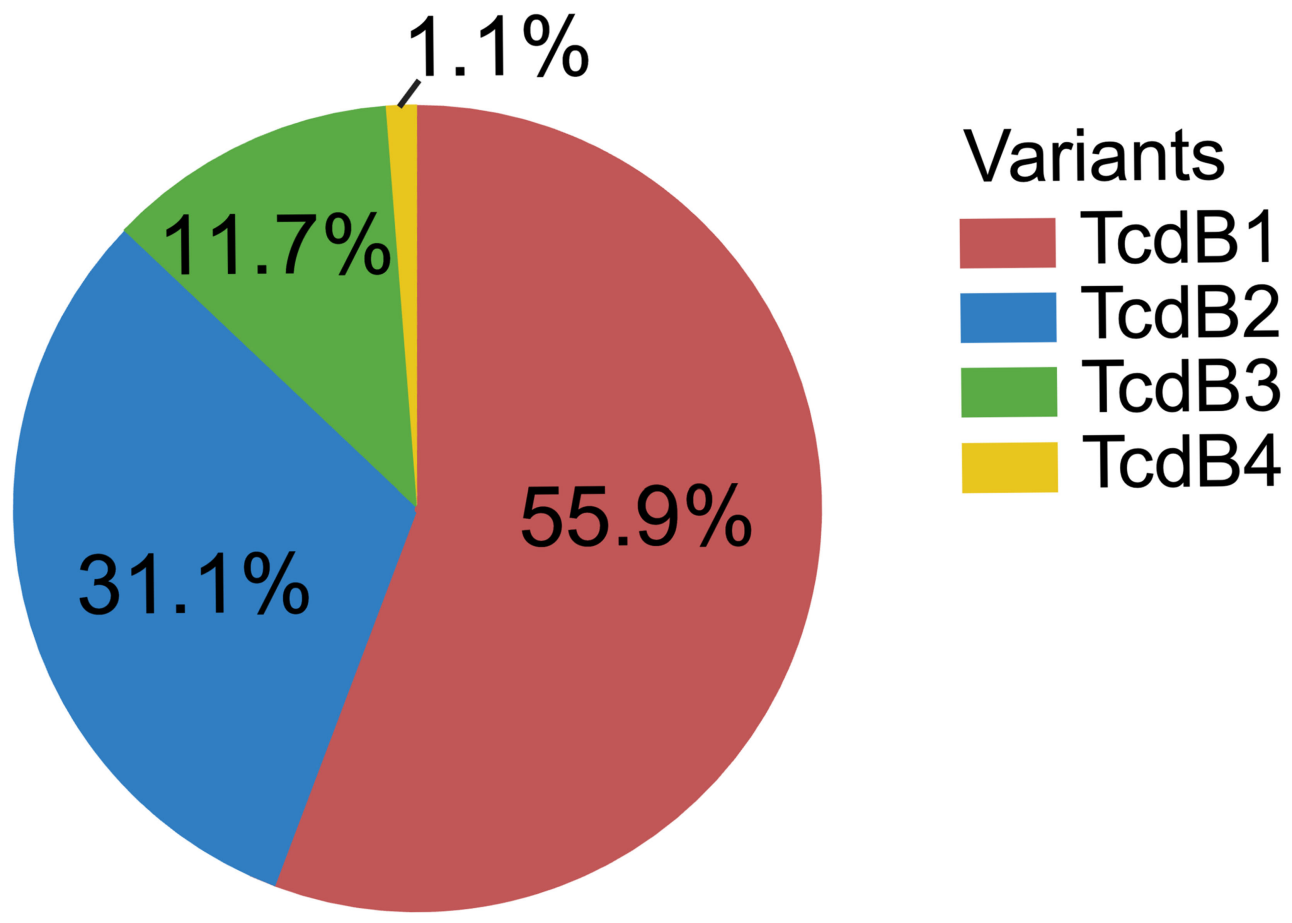
Distribution of major TcdB variants among global human isolates.

**Figure 2:**
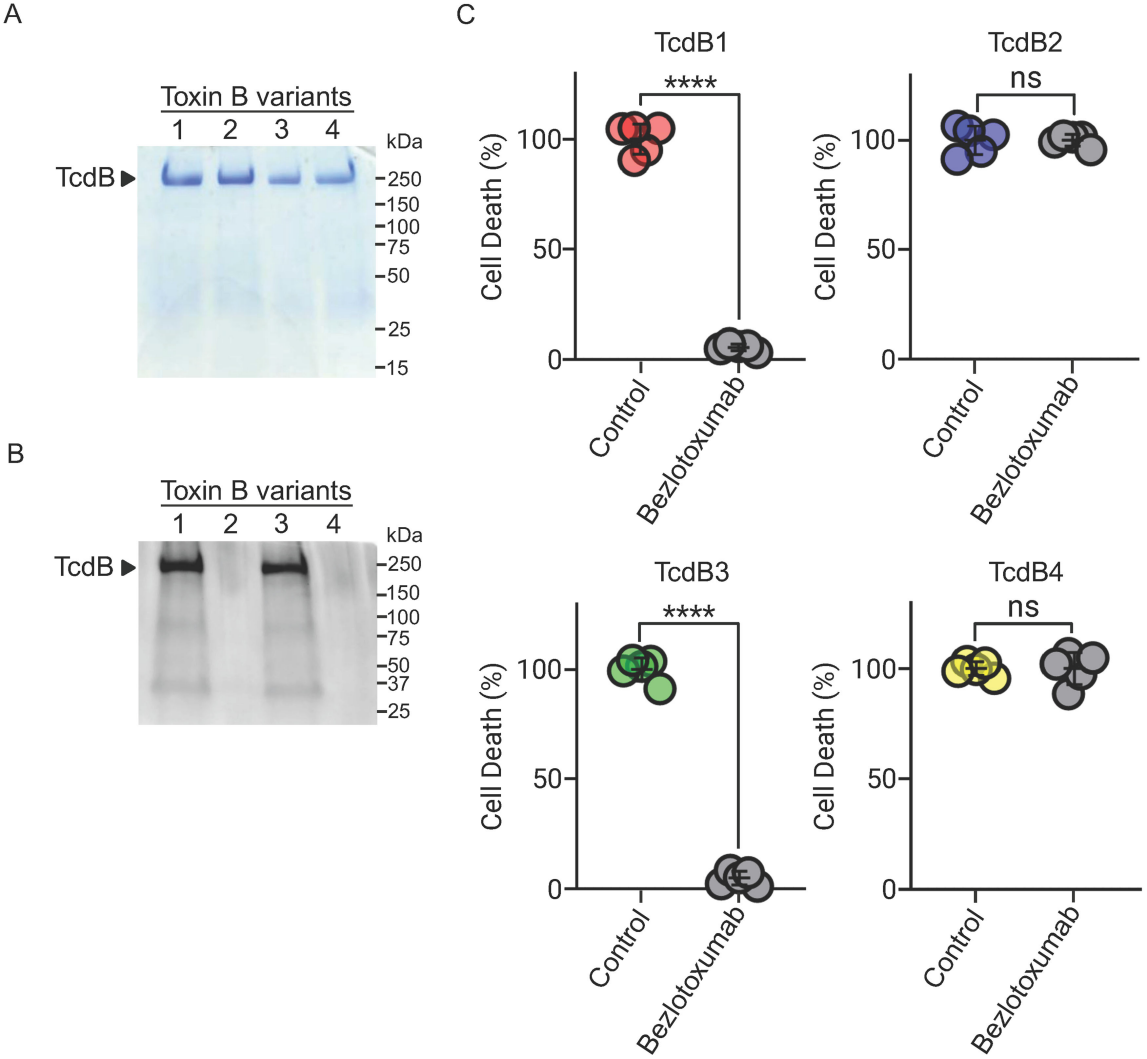
Toxin B (TcdB) variants differ in susceptibility to antibody-mediated neutralization. **(A, B)** Monoclonal antibody bezlotoxumab recognized TcdB1 and TcdB3 but not TcdB2 or TcdB4. SDS–PAGE analysis of purified TcdB1–TcdB4 variants **(A)** and immunoblot analysis of purified TcdB variants using bezlotoxumab **(B)**. **(C)** Cell death analysis of human colonic epithelial cells treated with TcdB variants in the presence of bezlotoxumab or control antibody (n = 5). Data represent the mean ± SD and are representative of 3 independent experiments. ****P <.0001. ns, not significant.

Given the significance of *C. difficile* infection worldwide, we sought to determine the global prevalence of TcdB variants. The analysis revealed that TcdB1 was the most prevalent variant (55.9%), followed by TcdB2 (31.1%), TcdB3 (11.7%), and TcdB4 (1.1%). Together, these four major variants accounted for approximately 99.9% of circulating TcdB in human isolates globally (**Figure 1**). Antibodies recognize epitopes on proteins that consist of specific amino acid sequences or residues. Studies have shown that bezlotoxumab has limited activity against the TcdB2 variant (Chen et al., 2021), raising the possibility that naturally occurring sequence variation in TcdB may limit antibody efficacy. Therefore, we assessed the activity of bezlotoxumab against the four major TcdB variants. Immunoblot analysis demonstrated that bezlotoxumab was able to recognize TcdB1 and TcdB3, but not TcdB2 or TcdB4 (**Figures 2A, B**). Consistent with these interaction findings, bezlotoxumab inhibited TcdB1 and TcdB3-mediated cell death in human colonic epithelial cells, but not TcdB2 and TcdB4 induced cell death (**Figure 2C**). Together, these findings suggest that sequence variation in TcdB determines susceptibility to antibody neutralization.

### Impact of amino acid substitution on antibody neutralization

**Figure 3:**
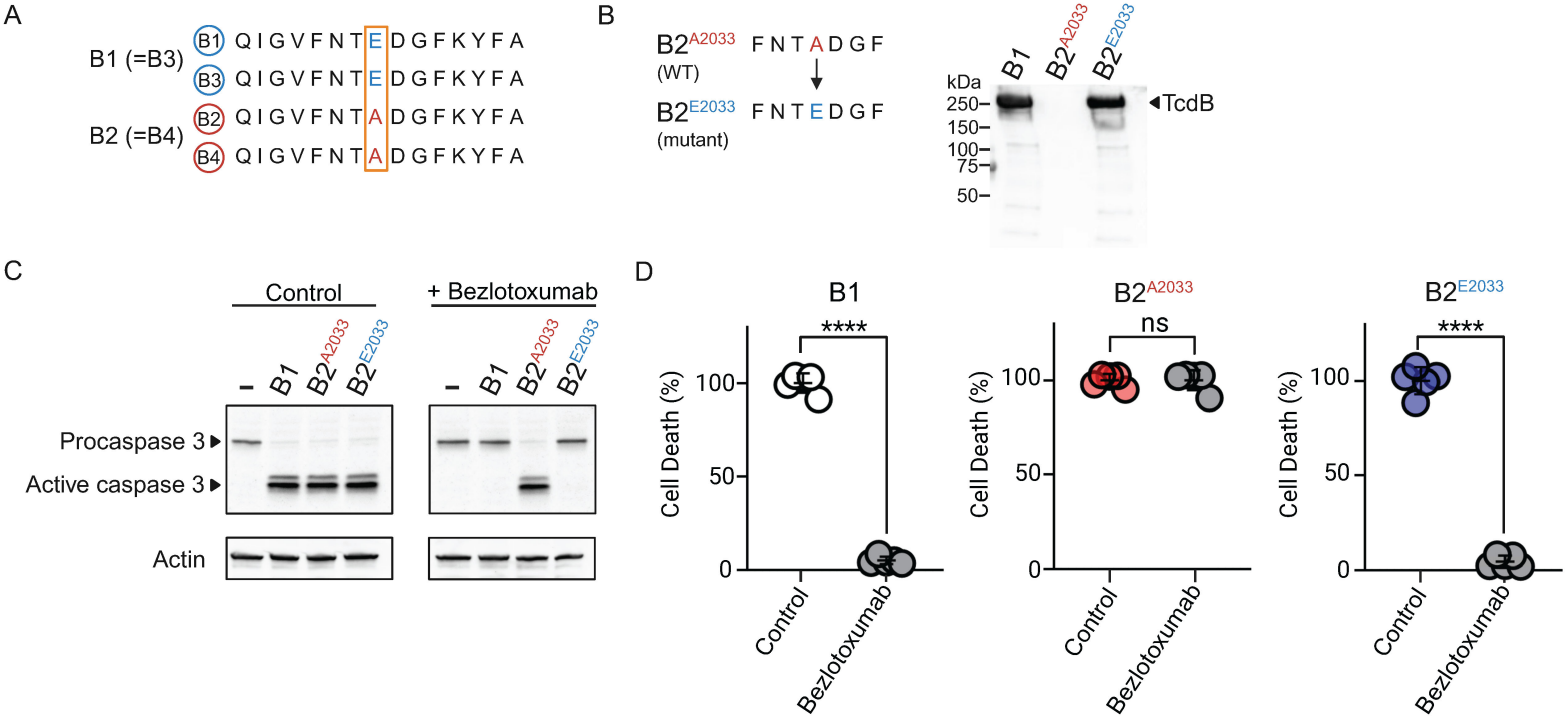
Effect of amino acid substitution on antibody neutralization. **(A)** Peptide sequence alignment of TcdB variants showing a single amino acid difference at position 2033, glutamate (E) in B1 and B3 versus alanine (A) in B2 and B4. **(B)** Schematic of the A2033E substitution in TcdB2 and immunoblot analysis of TcdB1, TcdB2, and the TcdB2 A2033E mutant using bezlotoxumab to assess antibody recognition. **(C, D)** A2033E substitution in TcdB2 restores antibody-mediated neutralization. Immunoblot analysis of activation of the cell death executioner caspase 3 **(C)** and evaluation of cell death **(D)** of human colonic epithelial cells treated with TcdB1, wild-type (WT) TcdB2 (A2033), or TcdB2 mutant (E2033) in the presence of bezlotoxumab or control antibody. Actin was used as a loading control. Cell death data represent the mean ± SD (n = 5). Data are representative of 3 independent experiments. ****P <.0001. ns, not significant; TcdB, *C. difficile* toxin B.

Single amino acid substitutions have been shown to influence susceptibility to antibody neutralization (Marone et al., 2025). Glutamate at position 2033 (E2033) of the TcdB protein has been identified in computational studies (Hernandez et al., 2015; Karnchanapandh et al., 2024), as well as our computational analysis (**Supplementary Figure 1**), as a potentially important residue in mediating interaction at a key bezlotoxumab epitope site. These analyses predict a favorable electrostatic interaction between TcdB residue E2033 and bezlotoxumab residue R59 (**Supplementary Figure 1**). TcdB1 and TcdB3 possess E2033, whereas TcdB2 and TcdB4 contain alanine at this position (A2033) (**Figures 3A, B**). This supports the hypothesis that E2033 is critical for bezlotoxumab neutralization. To test this hypothesis, we created a TcdB2 variant that possesses E2033 instead of A2033 (**Figure 3B**). Using immunoblot analysis, caspase activation, and cell death assays, we found that substitution of A2033 with E2033 in the TcdB2 variant restored susceptibility to antibody neutralization (**Figures 3C, D**). These findings demonstrate that a single amino acid substitution at position 2033 is sufficient to restore susceptibility to bezlotoxumab neutralization.

## Discussion

Despite the availability of fecal microbiota transplantation, antibiotics, and other therapies, the recurrence rates of *C. difficile* infection remain high, highlighting the need for alternative treatment strategies. Bezlotoxumab was approved for preventing recurrent *C. difficile* infection but was recently discontinued, further limiting treatment options and emphasizing the continued need for new therapies.

Monoclonal antibodies are a powerful therapeutic tool, offering highly specific targeting of epitopes. However, their high specificity can be a limitation, as even a single amino acid change in the target protein may significantly reduce efficacy (Nishimura et al., 2014; Marone et al., 2025). Understanding the mechanistic limitations of current antibodies, such as sequence-dependent neutralization, can inform the development of next-generation therapeutics. This is especially crucial for conditions like *C. difficile* infection because antibodies and vaccines are currently under development to treat and prevent infection (Alameh et al., 2024; Kroh et al., 2025; Peritore-Galve et al., 2025; Thomas et al., 2026). Our results demonstrate that the A2033E substitution in TcdB2 restores susceptibility to bezlotoxumab and suggests that variation at this residue may contribute to neutralization among TcdB variants. We found that TcdB sequence variation influences bezlotoxumab efficacy: the antibody neutralizes TcdB1 and TcdB3, which contain glutamate at position 2033, but not TcdB2 or TcdB4, and introducing E2033 into TcdB2 restores susceptibility, highlighting that a single amino acid can determine therapeutic effectiveness. Although TcdB4 was not tested with the E2033 substitution due to its low global prevalence (~1%), the TcdB2 experiments demonstrate that this single-residue change is sufficient to restore bezlotoxumab susceptibility, and a similar effect would be expected for TcdB4 based on sequence similarity. Our work provides mechanistic insight into how a single amino acid variation can drive antibody escape. This may also explain why AZD5148, a monoclonal antibody developed by AstraZeneca, exhibits reduced activity against TcdB3 *in vitro*, despite only a single amino acid substitution at the center of its epitope (Peritore-Galve et al., 2025). In addition to antibodies, peptide-based therapeutics are emerging as a potential alternative strategy to neutralize TcdB variants; however, it remains unclear to what extent they are affected by single amino acid substitutions. A recent study developed inhibitory peptides targeting a conserved region within the glucosyltransferase domain (GTD) with activity against TcdB1–3 (Catella et al., 2026). Given their short half-lives and susceptibility to degradation in the gastrointestinal tract, the ability to deliver peptide therapies safely and effectively to the gut will be key, as this might provide an additional therapeutic option (Sharma et al., 2025).

Understanding the limitations of currently available antibodies can aid in the development of therapeutics effective against *C. difficile*, regardless of the TcdB variant. There are ongoing efforts to explore new antibody and vaccine candidates (Alameh et al., 2024; Kroh et al., 2025; Peritore-Galve et al., 2025; Thomas et al., 2026), and naturally occurring variations in TcdB can significantly affect therapeutic efficacy. While a single monoclonal antibody may tolerate limited amino acid substitutions, even minor changes can result in loss of efficacy. Our findings suggest that such approaches should consider the development of broader antibodies, optimized combinations, and more clinically effective treatments that account for variant diversity. Alternatively, these findings support targeting highly conserved regions across variants, rather than regions susceptible to mutation or even a single amino acid substitution.

## Data Availability

The original contributions presented in the study are included in the article/**Supplementary Material**. Further inquiries can be directed to the corresponding author.
