# Supplementary material for: A single amino acid substitution determines susceptibility of Clostridioides difficile toxin B variants to monoclonal antibody neutralization
Source: Front Cell Infect Microbiol. 2026 May 28;16:1849926. doi: 10.3389/fcimb.2026.1849926 (PMC13253237; doi:10.3389/fcimb.2026.1849926)
Supplement: Supplementary file 1 [file DataSheet1.pdf]

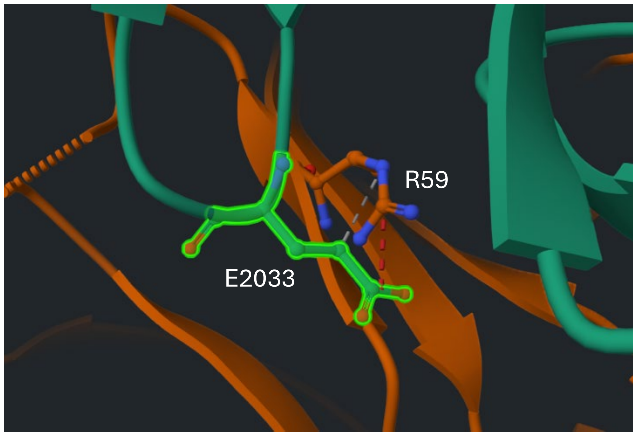

Supplementary Figure 1. Interaction between bezlotoxumab and toxin B (TcdB). Note the electrostatic (red dashed line) and van der Waals (grey dashed line) interactions between R59 of bezlotoxumab and E2033 of TcdB which are lost with A2033.
